# Post-Coma Neurorehabilitation: Neurophysiological Assessment as an Additional Strategic and Essential Competence for the Physiatrist

**DOI:** 10.3390/jpm15060260

**Published:** 2025-06-18

**Authors:** Luigi Di Lorenzo, Carmine D’Avanzo

**Affiliations:** Istituto Neurologico Mediterraneo Neuromed, Istituto di Ricovero e Cura a Carattere Scientifico, 86077 Pozzilli, IS, Italy

**Keywords:** neurorehabilitation, disorders of consciousness, neurophysiological assessment, post-coma recovery, physiatrist competence

## Abstract

Neurophysiological techniques, particularly somatosensory evoked potentials (SEPs) and electroencephalography (EEG), are essential tools for the functional and prognostic evaluation of patients with prolonged disorders of consciousness (DoC) in intensive neurorehabilitation settings. This narrative review critically analyzes the most relevant evidence regarding the use of SEPs and EEG in the management of post-comatose patients, highlighting the strategic role of physiatrists in integrating these assessments into individualized rehabilitation plans. A systematic search was conducted across major international databases (PubMed, Embase, Scopus, Cinahl, and DiTA) until December 2024, selecting consensus documents, official guidelines (including the 2021 ERC/ESICM guidelines), systematic reviews, observational studies, and significant Italian neurophysiological contributions. The literature supports the strong prognostic value of the bilateral presence of the N20 component in SEPs, while its early bilateral absence, particularly in post-anoxic cases, is a robust predictor of poor neurological outcomes. EEG provides complementary information, with continuous, reactive, and symmetrical patterns associated with favorable outcomes, while pathological patterns, such as burst suppression or isoelectric activity, predict a worse prognosis. Combining SEP and EEG assessments significantly improves prognostic sensitivity and specificity, especially in sedated or metabolically compromised patients. Additionally, the use of direct muscle stimulation (DMS) and nerve conduction studies enables accurate differentiation between central and peripheral impairments, which is crucial for effective rehabilitation planning. Overall, SEPs and EEG should be systematically incorporated into the evaluation and follow-up of DoC patients, and the acquisition of neurophysiological competencies by physiatrists represents a strategic priority for modern, effective, and personalized neurorehabilitation.

## 1. Introduction

In recent decades, numerous studies have highlighted the prognostic value of evoked potentials (EPs) and electroencephalography (EEG) in patients with severe acquired brain injury (ABI), whether of anoxic or traumatic origin. In both neurocritical and rehabilitation settings, these neurophysiological assessments have proven essential in supporting the differential diagnosis of disorders of consciousness (DoC) and contributing to the development of personalized rehabilitation plans [1,2,3,4,5]. The current literature, including international guidelines and position papers, highlights how cortical SEPs (particularly the N20 component) and EEG parameters (such as background continuity and the presence of reactive patterns) can serve as independent predictors of the potential for recovery of consciousness [6,7]. When competently and promptly integrated into the clinical rehabilitation process, these tools can guide critical neurorehabilitation and physiokinesiotherapy decisions, such as initiating collaborative robotic rehabilitation, continuation of ventilatory support, early initiation of intensive neuromotor and/or cognitive training, planning of neurophysiological follow-up, and family counseling [3,8,9].

In recent years, this evidence has progressively shaped the organizational and operational frameworks of modern neurorehabilitation. A significant expansion of specialized intensive neurorehabilitation units (classified in Italy as Code 75 MDC 1) has been observed throughout Europe, particularly in response to the increasing survival of patients with severe ABI following prolonged stays in intensive care units (ICUs). These patients often present with protracted alterations in consciousness, and the care continuum increasingly requires a coordinated transition from acute neurocritical management to subacute and long-term rehabilitation care. Within this clinical trajectory, neurophysiological methods originally developed and validated in ICUs are now being systematically translated and adopted in rehabilitation settings.

This transposition of knowledge has been facilitated by the growing integration of neurological disciplines, including neurology, clinical neurophysiology, rehabilitation medicine, and intensive care. Multidisciplinary collaboration, coupled with the development of shared clinical pathways, has enabled the transfer of critical monitoring and evaluation tools from ICUs to neurorehabilitation units. Notably, many of the same neurophysiological parameters initially used to stratify prognosis in the early stages of coma management have become cornerstones in assessing the recovery potential of patients with DoC, including those in the vegetative state (VS/UWS) or minimally conscious state (MCS).

The evolution of neurorehabilitation has also been supported by remarkable technological progress. Over the past decade, rehabilitation medicine has increasingly relied on advanced instrumental support—not only for treatment purposes (e.g., robotics, neuromodulation, and virtual reality) but also for diagnostic and prognostic functions. The availability of high-fidelity EEG systems, portable devices for SEP recording, and software platforms capable of longitudinal data integration have profoundly changed the way rehabilitation teams assess and follow patients with DoC.

When used within structured and dynamic care models, these tools allow for a more realistic, objective, and comprehensive formulation of the rehabilitation project. In particular, they help clinicians monitor the evolution of cortical and subcortical activity over time, which is crucial for guiding the rehabilitation plan during the weeks or months in which patients are treated within Code 75 units. Rather than relying solely on behavioral observation, physiatrists now have the opportunity to integrate electrophysiological data into their clinical reasoning, enabling earlier detection of neurofunctional changes, better adaptation of therapy intensity, and more accurate communication with patients’ families.

The integration of SEPs and EEG is particularly valuable when behavioral responsiveness is limited. In such cases, standardized neurobehavioral tools like the Coma Recovery Scale–Revised (CRS-R), developed by Giacino et al., play a critical role in identifying subtle signs of consciousness and guiding diagnostic classification. Combining the CRS-R with neurophysiological data enhances diagnostic and prognostic accuracy, especially in differentiating the vegetative state from the minimally conscious state [10].

Moreover, the integration of EPs and EEG in the evaluation of DoC aligns with the broader paradigm shift toward data-driven and personalized medicine in rehabilitation. As the clinical trajectories of patients with severe brain injuries become increasingly complex, there is a growing need for objective tools capable of providing reliable information on prognosis, cerebral reactivity, and residual brain function. These parameters are especially critical when clinical examination is limited by low responsiveness, pharmacological sedation, or the presence of secondary complications such as infections, hydrocephalus, or seizures.

The international scientific community has underscored the role of EPs and EEG not only as static indicators of prognosis but also as dynamic instruments for monitoring recovery. The presence of cortical responses (e.g., a preserved N20 component), the evolution of EEG background activity from discontinuous to continuous patterns, and the reappearance of reactivity to sensory stimuli have been correlated with transitions from VS/UWS to MCS and beyond. These observations are not merely of academic interest; they provide actionable insights that can directly influence rehabilitation planning, timing of interventions, and therapeutic intensity.

Furthermore, the inclusion of neurophysiological monitoring in the rehabilitation process reflects a growing appreciation of the complexity of recovery in patients with DoC. These patients often require months of care, during which subtle signs of change may indicate significant neuroplastic potential. In this context, serial EEGs and SEPs offer a valuable longitudinal view, complementing clinical scales such as the Coma Recovery Scale-Revised (CRS-R) and bridging the gaps between observed behavior and underlying cortical function.

Recent advances in electrophysiology, such as high-density EEG, somatosensory mismatch responses, and functional connectivity analyses, promise to further enhance the resolution and predictive value of neurophysiological data. While still under investigation, these techniques may soon complement standard EPs and EEGs, offering more refined assessments of thalamocortical integrity, stimulus-specific processing, and network-level dynamics, all of which are highly relevant to consciousness recovery and rehabilitation prognosis.

Taken together, these developments point to an increasingly central role of neurophysiology in rehabilitation medicine. Far from being confined to diagnostic moments, EPs and EEGs are now recognized as essential components of an ongoing evaluative framework that guides the entire rehabilitation process, from initial stratification to real-time adjustment of therapeutic goals. Their integration within the rehabilitation workflow is particularly important in Code 75 neurorehabilitation units, where patients with DoC are treated over extended periods and where clinical decision-making requires the highest degree of objectivity, timeliness, and personalization.

In this rapidly evolving context, physiatrists must expand their competencies and take an active role in interpreting and clinically applying neurophysiological findings. Promoting interdisciplinary collaboration with neurophysiologists, fostering training opportunities, and incorporating EP and EEG analyses into the standard evaluation toolkit can substantially improve the quality of care for patients with DoC.

Finally, the convergence of technological innovation, clinical evidence, and multidisciplinary collaboration provides a unique opportunity to redefine neurorehabilitation standards. As the field moves toward more precise, individualized, and outcome-oriented care models, the systematic use of EPs and EEGs is not merely a possibility but a necessity. Their integration into rehabilitative planning represents a pivotal advancement in ensuring that patients with severe brain injuries receive timely, accurate, and responsive interventions tailored to their evolving clinical status. This narrative review aimed to provide physiatrists with a clinically oriented synthesis of evidence on the use of SEPs and EEG in disorders of consciousness, with a focus on practical applications in neurorehabilitation.

## 2. Materials and Methods

To support the proposed framework, a narrative review of the literature was conducted to identify, synthesize, and critically analyze the most relevant evidence concerning the clinical and prognostic use of neurophysiological assessments—particularly somatosensory evoked potentials (SEPs) and electroencephalography (EEG)—in patients with disorders of consciousness (DoC), with specific attention to their integration in intensive neurorehabilitation settings.

The search strategy was defined a priori and carried out through a structured consultation of the principal international biomedical databases: PubMed, Embase, Scopus, Cinahl, and DiTA (Database of Interventions Tested in Applied Health Technology). The search was updated to include all relevant literature published until December 2024. No language restrictions were applied during the initial screening phase to ensure comprehensive sensitivity in the search process. The keywords and MeSH terms used in combination included: “somatosensory evoked potentials”, “SEP”, “EEG”, “coma”, “disorders of consciousness”, “rehabilitation”, “neurophysiology”, “post-anoxic”, and “post-traumatic brain injury”. Boolean operators (AND/OR) were used to ensure comprehensive article retrieval. As this is a narrative review, no systematic search or PRISMA flowchart was applied; instead, the selection prioritized clinical relevance and translational value for neurorehabilitation practice. Studies were selected based on their clinical relevance, publication in peer-reviewed journals, and inclusion of adult patients with DoC. We prioritized recent consensus statements and high-impact studies in the fields of neurorehabilitation and neurocritical care.

The inclusion criteria comprised clinical studies (randomized controlled trials, cohort studies, observational designs) addressing the use of EPs or EEG in DoC; Systematic reviews and meta-analyses; official guidelines and consensus statements from international scientific societies; and narrative reviews or expert opinions published in peer-reviewed journals with clear clinical implications and contributions by recognized neurophysiology experts relevant to the national context of neurorehabilitation.

The exclusion criteria were non-peer-reviewed abstracts, preprints without final peer-review outcomes, and studies that did not directly address DoC or rehabilitation outcomes.

In order to provide a balanced and context-specific overview, particular emphasis was placed on studies conducted in neurointensive care units, which serve as the origin of most prognostic neurophysiological frameworks. These data were then compared and integrated with studies and applications emerging within neurorehabilitation practice, especially in patients managed in Code 75 intensive neurorehabilitation units.

Consensus documents, such as the 2021 ERC/ESICM post-cardiac arrest guidelines, the Neurocritical Care Society 2024 recommendations, and the 2009 international consensus by Guérit et al. on neurophysiology in intensive care, were reviewed in full. These were complemented by major contributions from international neurophysiological literature, recognized for its longstanding tradition of electrophysiological monitoring in severe brain injuries, including seminal studies on SEP patterns, EEG classifications, and multimodal prognostic modeling.

Each reference was subjected to qualitative content analysis. The synthesis aimed not only to collect raw findings but also to critically contextualize them in light of the evolving role of the physiatrist in multidisciplinary rehabilitation teams. Additionally, cross-referencing between ICU-originated evidence and its application in neurorehabilitation settings was undertaken to delineate the trajectory of translational integration.

This methodological approach allowed for a multi-angle examination of the available literature and enabled the construction of a robust framework in which neurophysiological techniques are not only prognostic markers but also instruments for functional monitoring and rehabilitation planning. The final analysis thus presents a consolidated view of how SEP and EEG contribute to the longitudinal management of DoC, serving both diagnostic and therapeutic purposes within modern neurorehabilitation paradigms.

## 3. Results

The main selected studies are reported in Table 1, while Table 2 details the neurophysiological parameters assessed, study design, and key conclusions from the reviewed literature. The literature review enabled the selection of a significant number of studies and consensus documents supporting the integrated use of evoked potentials (EPs) and electroencephalography (EEG) in the functional and prognostic evaluation of post-comatose patients within the rehabilitation setting.

The main contributions, summarized in Table 1, highlight a direct correlation between the bilateral presence of the N20 component of somatosensory evoked potentials (SEPs) and the likelihood of recovery of consciousness in patients with prolonged disorders of consciousness [1,2,3,4,5,9].

One of the key reference studies, conducted by Moseby-Knappe et al., proposed a cortical N20 amplitude cut-off of <0.4 µV as a highly specific marker for poor neurological outcomes in post-cardiac arrest patients [4,5].

This recommendation has also been incorporated into the 2021 ERC/ESICM guidelines, which endorse its use as a second-level tool integrated with EEG and imaging modalities [6].

In parallel, EEG analysis has assumed a fundamental role in neurological risk stratification: a continuous, symmetrical, and reactive background is associated with a favorable prognosis, whereas patterns such as burst suppression, marked discontinuity, and isoelectric activity are predictive of poor outcomes [7,8].

The EEG classifications proposed by Synek and Hirsch, which have already been validated in the neurocritical care setting, also provide practical support during the rehabilitation phase by facilitating the clinical interpretation of EEG recordings [7,8,9].

Several authors, including Rossetti et al., have emphasized the effectiveness of the multimodal approach based on the integration of SEPs and EEG, which enhances prognostic sensitivity and specificity, particularly in patients under sedation or with altered metabolic conditions [2].

This integrated approach is crucial for minimizing false negatives and promptly guiding clinical and rehabilitation decisions.

Another relevant area that emerged from the review is the distinction between central and peripheral injuries, which is particularly important in patients with prolonged intensive care unit stays.

Santoro and Uncini [11], supported by targeted neurophysiological studies, outlined the characteristics of critical illness polyneuropathy and myopathy (CIP/CIM), highlighting the value of direct muscle stimulation (DMS) and distal potential analysis for the early identification of mixed patterns.

This approach enables a more accurate diagnostic definition in patients with generalized flaccid weakness and supports the development of an appropriate rehabilitation plan [11].

Taken together, this body of evidence confirms the strategic role of SEPs and EEG in supporting informed decision-making for neurorehabilitation.

**Figure 1:**
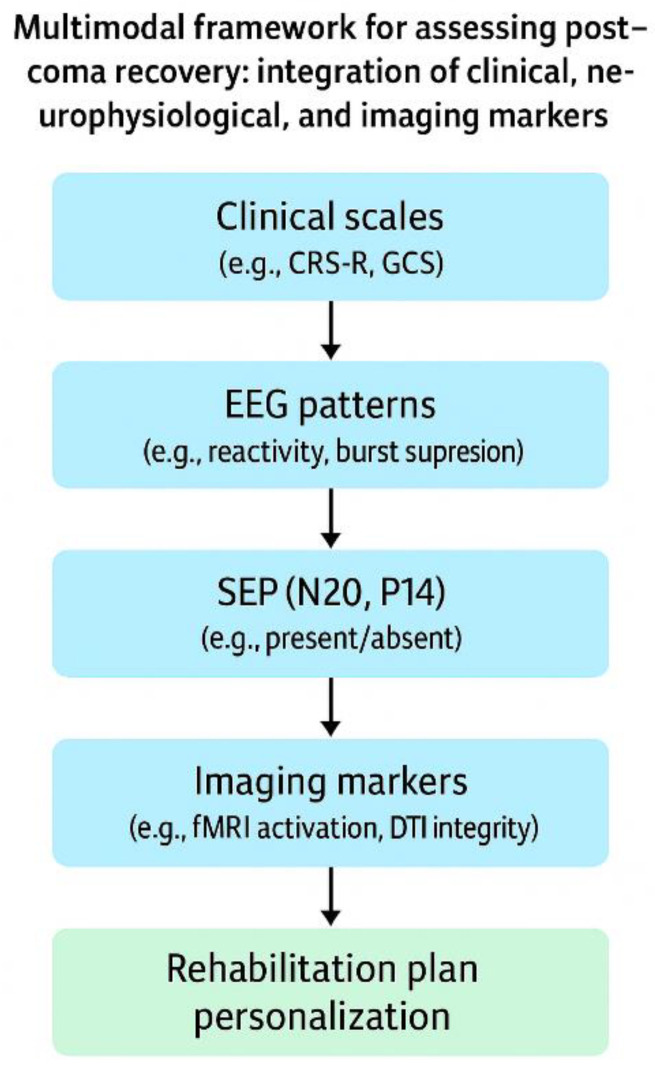
Multimodal framework for assessing post-coma recovery: integration of clinical, neurophysiological, and imaging markers.

The systematic use of these tools not only enables better prognostic stratification but also helps guide therapeutic interventions in a timely, individualized manner following procedure summarized done, Figure 1, facilitating shared decision-making with the healthcare team and the patient’s family (See Supplementary Table S1 for a stepwise summary of SEP and EEG integration in neurorehabilitation planning.) [1,2,3,4,5,6,7,8,9,11].

## 4. Discussion

In recent years, leading scholars and educators in clinical neurophysiology have systematically reported the most relevant evidence accumulated over the past two decades on the neurophysiological use of evoked potentials and other advanced electrophysiological techniques in neurocritical and intensive care settings. From this consolidated body of knowledge, several key protocols and translational evidence have emerged and are increasingly being adopted in neurorehabilitation. These experts consistently underline how such tools not only play a central role in early prognostic stratification but are also progressively becoming essential for clinical monitoring and longitudinal evaluation of functional recovery. The shared conclusions from these studies have contributed to the development of structured application protocols, which now extend into neurorehabilitation, where continuous monitoring of cortical and subcortical activity is fundamental for personalized therapeutic planning.

At a recent symposium held under the aegis of the Chair of Neurophysiology at the University of Naples, chaired by Prof. Lucio Santoro and with the contribution of Prof. Aldo Amantini, the latter effectively summarized the core scientific evidence currently guiding clinical decision-making. Among the most consistent findings, one of the strongest is the predictive value of somatosensory evoked potentials (SEPs) and electroencephalography (EEG) in the early stages after acute neurological injury, with direct implications for rehabilitation planning. An international consensus statement by Guérit et al. [12] established the clinical utility of EEG, SEPs, and ENMG in the intensive care unit, formally recognizing these as core tools in integrated neurophysiological evaluation. Specifically, median nerve SEPs—particularly the components Erb’s point, N13, P14, and N20—serve as objective markers of central somatosensory integrity, especially within the parietal cortex (area 3b). A comparison between EEG and SEP has further revealed their distinct diagnostic properties: EEG is more vulnerable to pharmacological sedation and metabolic disturbances, whereas SEPs demonstrate greater reliability in cases of structural hypoxic-ischemic injury with improved standardization and interpretability [13]. The classification introduced by Houlden et al. [14], which identifies six bilateral SEP patterns (from absent-absent to normal-normal), showed that the AA pattern was strongly associated with poor prognosis, while the NN pattern was predictive of functional recovery. These observations have been confirmed by later studies, including that of Wadiura et al. [15], who introduced a visual SEP scoring system that enhanced its clinical utility. Amantini et al. [16], through ROC curve analyses, demonstrated that bilaterally normal or asymmetrically abnormal SEP patterns are significantly associated with awakening and reduced disability. Similarly, other authors [17], in a systematic review, concluded that SEPs, while not without limitations, are the most accurate isolated predictors of outcome in patients with severe brain injury, outperforming traditional clinical markers such as GCS, EEG, and pupillary reactivity.

Hirsch LJ [18] highlighted the strong correlation between SEP presence/quality and the probability of awakening across various etiologies of coma, showing that bilateral SEP absence is almost invariably associated with poor outcomes in both post-anoxic and traumatic conditions. Reinforcing this view, it has been reported [6,19,20] that in the presence of high-quality SEP recordings, the bilateral absence of the N20 component is a highly reliable marker of poor neurological outcomes following cardiac arrest. Aalberts [21] further supported this by noting that even in the therapeutic hypothermia era, the prognostic value of absent N20 remains solid and that the few published exceptions are methodologically flawed and insufficient to undermine the predictive strength of SEPs.

Within the context of traumatic brain injury, the prognostic role of SEPs has been reinforced by several key studies. Houlden et al. [14], in a large cohort, showed that 96% of patients with bilaterally normal SEPs (grade 6) achieved functional independence at one-year follow-up. Other authors [22] compared ROC curves for SEPs and GCS, concluding that SEPs offer superior prognostic accuracy in predicting the transition to minimally conscious states. Bagnato et al. [23] demonstrated a strong correlation between SEP amplitude and recovery of consciousness in patients with unresponsive wakefulness syndrome (UWS), making it a valuable objective index of neurological severity and potential for rehabilitation. Finally, the recent 2024 guidelines from the Neurocritical Care Society [24] emphasized that TBI severity, traditionally assessed using the GCS, may be more accurately defined when integrated with neurophysiological markers such as SEPs.

Taken together, these findings provide a methodologically sound and statistically robust case for the systematic inclusion of SEPs in the early post-acute phase of care, supporting more realistic prognostic assessments, better risk stratification, and timely rehabilitation planning tailored to individual patients.

In recent years, the field of neurorehabilitation has definitely undergone significant acceleration at the organizational, technological, and methodological levels, with tangible effects on clinical practice. The global expansion of Neurorehabilitation Units (classified in Italy as Code 75), now commonly integrated within Neuroscience Departments, has benefited from the expertise and collaborative input of intensivists and clinical neurophysiologists, as previously noted. Non-invasive neuromodulation techniques, such as transcranial magnetic stimulation (TMS) and transcranial direct current stimulation (tDCS), are increasingly being applied in the treatment of stroke, traumatic brain injury (TBI), and neurodegenerative diseases [25].

At the same time, invasive techniques such as spinal cord stimulation (SCS) and vagus nerve stimulation (VNS) are gaining traction in managing chronic pain and promoting motor recovery after central nervous system lesions [26]. The introduction of robotic systems and intelligent exoskeletons has further contributed to reshaping the rehabilitative approach, offering adaptive, AI-driven platforms capable of personalizing motor training. When combined with neuroplasticity-enhancing strategies alongside task-specific training—these approaches have shown promising results in extending the optimal recovery window, particularly in the subacute post-injury phase.

The use of immersive virtual reality (VR) platforms and the application of gamification principles have emerged as powerful tools for enhancing patient motivation, adherence, and movement repetition, which are critical for driving adaptive cortical reorganization. On the neurotechnology front, brain-computer interfaces (BCIs) are creating new possibilities for decoding motor intention and facilitating real-time neural modulation, often in synergy with exoskeletons or functional electrical stimulation (FES) systems [27].

In parallel, growing attention is being paid to the identification of advanced neurophysiological and neuroimaging biomarkers—such as functional MRI (fMRI), diffusion tensor imaging (DTI) [28], and blood-based markers (e.g., neurofilament light chain)—to support treatment personalization and longitudinal monitoring of neurological recovery.

Simultaneously, the adoption of digital health tools for remote monitoring has become increasingly widespread. Wearable sensors and mobile applications now enable continuous data collection on muscle strength, mobility, and participation, thereby supporting the development of adaptive telerehabilitation models [29,30,31,32].

Within this rapidly evolving landscape, applied neurophysiology is attracting growing interest in the Neurorehabilitation Unit, particularly through tools such as evoked potentials (EPs) and electroencephalography (EEG), which serve as an essential bridge between objective functional assessment and individualized rehabilitation planning. Building on this foundation, an increasing number of studies have explored how these neurophysiological tools—originally developed for intensive care settings—are now being integrated into neurorehabilitation protocols, offering valuable prognostic and monitoring functions throughout the recovery continuum.

Neurophysiological assessment through evoked potentials and EEG recordings has indeed assumed an increasingly important role in the clinical management and prognostic evaluation of patients with prolonged disorders of consciousness in neurorehabilitation. These tools allow for an objective measurement of functional pathways, even in the absence of behavioral responsiveness, and are fundamental in the development of realistic and personalized rehabilitation programs.

In particular, analyzing the selected papers reported in Table 2, we found that in clinical practice, the presence of the N20 component in SEPs serves as a robust marker of somatosensory integrity, while its early bilateral absence is strongly predictive of a poor prognosis, as consistently demonstrated by multiple studies [1,2,3,4,5].

In parallel, EEG provides complementary information, with favorable patterns associated with better recovery outcomes and disorganized or isoelectric patterns correlated with poor outcomes [5,6].

The integrated EEG + SEP approach, also endorsed by European guidelines [6], ensures greater accuracy in prognostic stratification.

Professor Amantini emphasized the importance of longitudinal SEP monitoring in patients with Unresponsive Wakefulness Syndrome (UWS), which is useful for tracking neurophysiological evolution over time [3].

Finally, the distinction between central and peripheral injury in hypotonic or flaccid patients following prolonged ICU stays is supported by the work of Santoro and Uncini [11] through the application of direct muscle stimulation techniques and nerve conduction analysis.

The ability to objectively measure the functionality of somatosensory, visual, motor, and auditory pathways, even in the absence of overt behavioral responses, has made these tools indispensable for physiatrists in developing personalized rehabilitation programs.

The integration of neurophysiological assessments with clinical observation allows practitioners to overcome the limitations of traditional neurological examinations, which are often complicated by sedation, fluctuating levels of consciousness, or severe motor deficits.

Numerous studies, confirmed by meta-analyses and international guidelines, have demonstrated that the presence of the cortical N20 potential in SEPs is strongly predictive of favorable outcomes in post-comatose patients.

Conversely, its bilateral absence, particularly in post-anoxic cases, represents one of the most robust markers of poor neurological outcomes.

Studies such as that by Moseby-Knappe et al. have even identified amplitude thresholds (below 0.4 µV) as cut-off values for the definition of poor prognosis, contributing to greater precision in risk stratification [5].

These findings have been widely confirmed in the 2021 ERC-ESICM guidelines [6], which recommend the systematic inclusion of SEPs in the early prognostic evaluation following cardiac arrest.

In contrast, EEG serves as a complementary but not a substitutive tool.

A continuous and reactive background, interhemispheric symmetry, and absence of pathological patterns, such as burst suppression or isoelectric activity, are associated with favorable outcomes.

However, EEG interpretation can be hindered by pharmacological, metabolic, and environmental interference.

In this context, the synergistic use of SEPs and EEG significantly enhances predictive sensitivity and specificity, as demonstrated by Rossetti et al. and other authors [1,2].

The classification proposed by Synek [8] and the more recent one by Kilbride [7] have further standardized EEG interpretation, providing tools applicable even in rehabilitation settings.

A particularly significant contribution comes from the Italian school, especially from the work of Professor Aldo Amantini, who promoted the use of SEPs not only for prognostic purposes but also as a tool for longitudinal monitoring [3].

The ability to detect qualitative and quantitative changes over time, even in the absence of apparent clinical modifications, allows physiatrists to adjust therapeutic strategies dynamically and realistically.

The absence of progression in neurophysiological recordings or the worsening of cortical responses may serve as indicators of recovery stagnation or the need to reassess treatment goals and intensity.

Equally important is the role of neurophysiology in supporting the differential diagnosis of central and peripheral conditions.

In particular, for patients who remain hypotonic or uncooperative after prolonged ICU stays, it is crucial to investigate the potential presence of critical illness polyneuropathy (CIP) or critical illness myopathy (CIM).

Santoro and Uncini [11] highlighted how alterations at the paranodal junction and secondary axonal damage, which are typical of these conditions, can be studied through targeted examinations such as direct muscle stimulation (DMS) and the assessment of residual conduction times.

This approach helps avoid interpretative errors and allows differentiation between irreversible cortical deficits and potentially reversible peripheral disconnection.

Overall, the results of the literature converge toward a clear recommendation: evoked potentials and EEG should be considered routine tools in the evaluation and follow-up of patients with disorders of consciousness. Finally, it is important to acknowledge that the implementation of SEP and EEG in clinical neurorehabilitation faces several challenges, including the influence of sedation, potential artifacts in suboptimal recording environments, and the need for interpretation by trained neurophysiologists. These limitations must be considered when integrating these tools into routine clinical practice.

Their adoption not only improves prognostic and diagnostic accuracy but also optimizes resource utilization, reduces clinical uncertainty, and better supports families in decision-making.

For physiatrists, becoming proficient in these tools means assuming an increasingly central role in the care pathway, oriented toward personalization, accountability, and timely intervention.

## 5. Conclusions

Neurophysiological techniques, particularly somatosensory evoked potentials (SEPs) and electroencephalography (EEG) are essential tools for the functional and prognostic evaluation of patients with prolonged disorders of consciousness admitted to intensive neurorehabilitation units (classified as code 75 MDC 1 in Italy).

Their interpretation, when integrated into a structured clinical rehabilitation model, allows practitioners to overcome the limitations of traditional neurological examinations and strengthen the decision-making capacity of the rehabilitation team.

In this context, the physiatrist assumes a central role not only in prognostic stratification and defining therapeutic goals but also in managing communication with families and dynamically adjusting treatment strategies over time.

Promoting the systematic use of these techniques and, above all, encouraging the acquisition of neurophysiological expertise among physiatrists represents a strategic priority for modern neurorehabilitation, aiming for expertise, effectiveness, and personalization of interventions.

## Figures and Tables

**Table 1:** Selected studies on SEP, EEG, and neurological outcomes.

Author	Year	Study Type	Focus
Rossetti AO et al.	2020	MRCT	EEG D.O.C [1]
Rossetti AO et al.	2017	Revision	SEP + EEG in DoC [2]
Amantini A et al.	2019	Observational	SEP e prognosi in UWS/MCS [3]
Baars JH et al.	2017	Clinical Study	SEP monitoring—N20 [4]
Moseby-Knappe M Backman S et al.	2020	Prospective Study	SEP N20 < 0.4 µV post-anossia [5]
Nolan JP et al., ERC/ESICM Guidelines	2021	Guidelines	SEP + EEG in prognosi post-rianimazione [6]
Kilbride RD, Hirsch LJ et al.	2013	Classification EEG	EEG grading e outcome [7]
Synek VM	1988	EEG prognostic	Classificazione EEG coma traumatico/anossico [8]
Gosseries et al.	2011	Clinical Study	SEP e recovery of consciousness [9]
Santoro L, Uncini A	2020	Revision	Elettrofisiologia nelle polineuropatie [11]
Soelaeman et al.	2021	Consensus	DMS, CIP/CIM, pattern miopatici [12]

**Table 2:** Key Selected studies: Neurophysiological parameters and prognostic value.

Authors	Year	Assessed Parameter	Conclusions	Level of Evidence
Moseby-Knappe M Backman et al.	2020	SEP—N20 (amplitude < 0.4 µV)	Highly predictive of poor neurological outcome after cardiac arrest	High [5]
Rossetti et al.	2017	SEP + EEG combined	Increases predictive sensitivity and specificity compared to individual tests.	High [2]
Amantini et al.	2009	SEP—N20, follow-up SEP	Present SEPs predict potential recovery and are useful for monitoring	Good [3]
Synek VM	1988	EEG—pattern prognostic	Isoelectric activity and burst suppression are associated with poor outcome	Seminal paper [8]
Kilbride RD, Hirsch LJ et al.	2013	EEG—reactivity and background	Reactive and continuous EEG pattern is associated with a favorable outcome	good [7]
Uncini & Santoro	2020	DMS, neuroxons CIP	Paranodal injury mechanism; essential to distinguish from axonal damage centrale vs. periferico	High [11]
ERC/ESICM Guidelines	2021	SEP, EEG	Recommended for early prognostication after anoxic injury	Evidence A [6]

Legend (Table Columns): Assessed Parameter: Main neurophysiological technique or signal analyzed in the study. Conclusions: Summary of the evidence reported in relation to prognosis in Disorders of Consciousness (DoC). Level of Evidence: Based on methodological robustness and clinical relevance, numbers in parentheses refer to the previously provided reference numbers.

## Data Availability

No new data were created or analyzed in this study.

## Supplementary Materials

The following supporting information can be downloaded at: https://www.mdpi.com/article/10.3390/jpm15060260/s1, Table S1: Stepwise integration of SEP and EEG findings in neurorehabilitation planning for patients with disorders of consciousness.
