# Supplementary material for: Post-Coma Neurorehabilitation: Neurophysiological Assessment as an Additional Strategic and Essential Competence for the Physiatrist
Source: J Pers Med. 2025 Jun 18;15(6):260. doi: 10.3390/jpm15060260 (PMC12194344; doi:10.3390/jpm15060260)
Supplement: Supplementary file 1 [file jpm-15-00260-s001.zip › jpm-3644482-supplementary.pdf]

Table S1. Stepwise integration of SEP and EEG findings in neurorehabilitation planning for patients with disorders of consciousness.

Step	Assessment	Interpretation	Clinical Action
1	Baseline EEG	Continuous background, symmetry, reactivity	Consider early rehab intensification
2	SEP – N20 component	Present bilaterally	Good prognosis; initiate active stimulation
3	SEP – N20 absent bilaterally	Poor prognosis	Discuss realistic goals with family; reassess intensity
4	Longitudinal EEG/SEP	Changes over time	Adapt therapy intensity and targets
5	Correlation with clinical status	Consistency with CRS-R or behavioral scales	Update rehab plan accordingly
6	Combine with neuroimaging	DTI/fMRI match SEP/EEG data	Refine personalized trajectory
